# Minimum dietary diversity and associated factors among children aged 6–23 months in Addis Ababa, Ethiopia

**DOI:** 10.1186/s12939-017-0680-1

**Published:** 2017-10-12

**Authors:** Dagmawit Solomon, Zewdie Aderaw, Teketo Kassaw Tegegne

**Affiliations:** 1 GAMBY College of Medical Science, Addis Ababa, Ethiopia; 2 Department of Public Health, College of Medicine and Health Sciences, Debre Markos University, Debre Markos, Ethiopia

**Keywords:** Minimum dietary diversity, Infant and young child feeding practice, Food groups, Ethiopia

## Abstract

**Background:**

Dietary diversity has long been recognized as a key element of high quality diets. Minimum Dietary Diversity (MDD) is the consumption of four or more food groups from the seven food groups. Globally, only few children are receiving nutritionally adequate and diversified foods. More than two-thirds of malnutrition related child deaths are associated with inappropriate feeding practice during the first two years of life. In Ethiopia, only 7 % of children age 6–23 months had received the minimum acceptable diet. Therefore, the main aim of this study was to determine the level of minimum dietary diversity practice and identify the associated factors among children aged 6–23 months in Addis Ababa, Ethiopia.

**Methods:**

A health facility based cross sectional study was undertaken in the three sub-cities of Addis Ababa from 26th February to 28th April, 2016. A multi-stage sampling technique was used to sample the 352 study participants or mothers who had children aged 6–23 months. Data were collected by using a structured and pretested questionnaire, cleaned and entered into Epi info 7 and analyzed using SPSS 24 software. Logistic regression was fitted and odds ratio with 95% confidence interval (CI) with *p*-value less than 0.05 was used to identify factors associated with minimum dietary diversity.

**Result:**

In this study, the overall children with minimum dietary diversity score were found to be 59.9%. Mother’s educational attainment and a higher household monthly income were positively associated with the minimum dietary diversity practice. Similarly, mothers’ knowledge on dietary diversity and child feeding was positively associated with minimum dietary diversity child feeding practice, with an adjusted odds ratio of 1.98 (95% CI: 1.11–3.53).

**Conclusion:**

In this study, the consumption of minimum dietary diversity was found to be high. In spite of this, more efforts need to be done to achieve the recommended minimum dietary diversity intake for all children aged between 6 and 23 months.

## Background

The World Health Organization (WHO) has established guidelines with respect to Infant and Young Child Feeding (IYCF) practices for children aged 6–23 months by considering Minimum Dietary Diversity (MDD) as one of the core eight indicators [1]. *“Minimum Dietary Diversity is the consumption of four or more food groups from the seven food groups for higher dietary quality and to meet daily energy and nutrient requirements of the seven recommended food groups namely: grains, roots and tubers; legumes and nuts; dairy products; flesh foods (meat, fish, poultry and organ meats); eggs; vitamin-A rich fruits and vegetables; other fruits and vegetables”* [2]. This cut–off point was used due to its association with a better quality diet both for breastfed and non-breastfed children [3]. “*Consumption of foods from at least four food groups on the previous day would mean that in most populations the child had a high likelihood of consuming at least one animal-source food and at least one fruit or vegetable that day, in addition to a staple food (grain, root or tuber)”* [2].

Dietary Diversity (DD) is a major factor for all people to meet the requirements for essential nutrients. Improved feeding practices by provision of adequately diversified food can lead to improved intake of energy and nutrients, which leads to better nutritional status. In contrary, inappropriate feeding practice is one of the reasons for under nutrition in many developing countries where diets are mostly based on starchy staples (staple foods) and seasonal fruits and vegetables with few or no animal products [1].

Globally, only a few children are receiving nutritionally adequate and diversified foods. In many countries, less than one fourth of infants aged 6–23 months meet the criteria for dietary diversity and feeding frequency [4]. Inappropriate complementary feeding practices increase the risk of under nutrition, illness and mortality among children under the age of two years [5, 6]. Children who do not receive sufficient dietary diversity and meal frequency after 6 month of age become stunted, despite they have optimum breastfeeding [7]. In 2015, according to WHO, 156, 50 and 42 million of the world under-five children were estimated to be stunted, wasted and overweight or obese respectively. About 45% of the global child deaths are associated with undernutrition [4]. More than two-thirds of malnutrition related child deaths are associated with inappropriate feeding practice during the first two years of life [5, 6].

The age of a child between 6 and 23 months is the critical windows of opportunity to prevent childhood malnutrition, and is a period of growth faltering and malnutrition since children need more energy and nutrient dense foods to grow and develop [1, 2, 8]. There will be frequent childhood illness like diarrheal diseases and infections as well as high nutrient requirement in addition to breast milk to sustain normal development [8]. Moreover, nutritional deficiencies during this period can lead to impaired cognitive development, growth retardation, smaller adult stature, and a consequence of compromised educational achievement and low economic productivity which become impossible to reverse later in life [2, 8, 9]. Therefore, during this period, proper infant and young child feeding practice; that is, appropriate, safe, adequate and frequent child feeding is important for the optimal growth of a child, better health and development [10].

In Ethiopia, according to the 2016 Ethiopian Demographic and Health Survey, 38.4% of children are stunted, 9.9% are wasted, and 23.6% are underweight indicating the persistence of both acute and chronic under nutrition [11]. Moreover, only 7 % of the children have been fed the minimum acceptable diet [11]. Detailed analysis of the 2011 Ethiopian Demographic and Health Survey showed that 10.8% and 44.7% of children aged 6–23 months have received minimum dietary diversity and minimum meal frequency respectively [12]. Similarly, three community-based studies done in the country showed that the minimum dietary diversity were 10.6% [13], 12.6% [14] and 17.8 [15]. Furthermore, two studies done in Ghana and India found that the minimum dietary diversity consumption in the previous one day were 35.3% [16] and 32.6% [17], respectively.

Minimum dietary diversity was significantly associated with mothers education [12, 14], wealth quintile [12], urban residence, home gardening and media exposure [14]. It was found that a family who grew vegetables and own livestock, a woman who received education on IYCF during postnatal care visits, maternal knowledge of IYCF, and exposure to IYCF information on mass-media were significantly associated with an increased in dietary diversity [13]. Having postnatal care visit and mother’s education were significantly associated with appropriate complementary feeding [15]. Similarly, a significant association of receiving appropriate complementary feeding was observed among children who were not bottle-fed in the previous one day [16].

However, the above-mentioned studies gave important information on infant and young child feeding practices, they had some limitations. The two studies done in Ethiopia [12, 14] didn’t see the influence of caregivers knowledge of infant and young child feeding on children minimum dietary diversity consumption. The other study, done in Southern Ethiopia [13], didn’t give a particular emphasis on minimum dietary diversity. The factor analysis was done for dietary diversity using linear regression. Moreover, assessment of maternal knowledge of IYCF had measurement errors. Mothers were considered as knowledgeable if they knew two of the four measurement parameters; that is a timely initiation of family food, a timely initiation of complementary food, dietary diversity and duration of breastfeeding. The study didn’t put a clear definition of each measurement. Similarly, the two studies done in Ethiopia [15] and Ghana [16] didn’t explore the factors associated with minimum dietary diversity rather a focus was given for appropriate complementary feeding. Furthermore, the study reported maternal knowledge and attitude of IYCF; however, there was no detail description how these were measured [15]. The last but not the least, two studies had a problem of sample size determination [15] and selection bias [17].

Generally, in Ethiopia the nutritional quality of foods offered to children is often poor when compared to nutritional requirements. Dietary diversity practice in urban area are often thought to be better, however, in Addis Ababa, the proportion of minimum dietary diversity practice is low [18]. In spite of dietary diversity being a major determinant to meet requirements for essential nutrient, which intern leads to better nutritional status, different researches conducted on IYCF emphasized on timely initiation of breast-feeding and introduction of complementary food. Even though there were researches done on dietary diversity and meal frequency, they were community-based studies done in rural areas of Ethiopia and they had also some methodological limitations as mentioned above. Therefore, the aim of this study was to assess minimum dietary diversity and the associated factors among children aged 6–23 months in Addis Ababa.

## Methods

### Study design and population

Health facility based cross-sectional study was employed to assess minimum dietary diversity and associated factors among children aged 6–23 months in Addis Ababa, Ethiopia from 26th February to 28th April, 2016. Addis Ababa lies between 2200 and 2500 m above sea level and serve as a home city for African union. The city comprises 10 sub-cities (Kifle Ketema) with 104 Kebeles (the lowest administrative unities in Ethiopia).

Based on the 2007 census, Addis Ababa has a total population of 2,738,248, where 36,443 and 195,932 of them are infants and under-five children respectively. In the city 662,728 households were counted living in 628,984 housing unit. Although all Ethiopian ethnic groups are represented in Addis Ababa due to its position as a capital city of the country, the two largest groups are Amhara (47.04%) and Oromo (19.51%). The religion with the most believers in city is Ethiopian Orthodox Christians with 74.7% of the population, while 16.2% are Muslim, 7.77% Protestant, and 0.48% Catholic [19]. There are 93 health centers and 6 hospitals administered by the Addis Ababa Health Bureau providing health services.

All children aged 6–23 months along with their mothers/caregivers who came for health services at governmental health centers in Addis Ababa, Ethiopia were the source populations. The study populations were all children of age 6–23 months along with their mothers/caregivers who came for health services to the Maternal and Child Health care (MCH) clinic for Expanded Program on Immunization (EPI) at the randomly selected governmental health centers in the three sub-cities of Addis Ababa, Ethiopia. In this study, to minimize under estimation due to loss of appetite, those children who were sick during the previous one week were excluded. Furthermore, even though it is a rare event in Ethiopia, children who came with their fathers or other than caregivers/mothers were excluded from the study, as they might not provide correct information on their dietary diversity or feeding practices.

### Sampling

A single population proportion formula was used to determine the sample size considering the following assumptions: 95% confidence level, 5% margin of error, 12.3% proportion of children with adequate dietary diversity in Addis Ababa [18], a design effect of two and non-response of 10%. This gave a sample size of 365; however, only 352 of the children aged 6–23 months were participated in this study.

A multi stage sampling technique was used to select the study participants. At stage one, from 10 sub-cities; assuming homogeneity, the three sub-cities (30%) were selected randomly using a lottery method. Then, at stage two, from the 25 health centers in the three sub-cities, eight health centers were selected by simple random sampling. For each health center the sample size was allocated proportional to the total number of children aged 6–23 month who came for health services; that is, from 26th February to 28th April, 2016, to the MCH clinic for EPI. Finally, the study participants were identified by using a systematic random sampling method. The sampling interval K was determined using the number of the flow of children to the selected health centers in relation to the allocated sample size for each health center. The first respondent from each health center was chosen using the lottery method and the subsequent respondents were determined by the sampling interval (every K^th^).

### Data collection/measurement and quality control

A structured pretested questionnaire was used to collect the required quantitative information through face-to-face interview with child’s mothers/caregivers. It was first prepared in English and then translated to Amharic (local language), then again back to English to check its consistency. The two investigators made the translation independently and came together to manage some inconsistencies. It was focused on socio-demographic characteristics of children, obstetrics and health service characteristics of mothers, knowledge of mothers on dietary diversity and child feeding, and dietary diversity feeding practices. The data collection tool regarding the various factors was adapted from the 2011 EDHS [18] with some modification to fit with the context. Moreover, data on dietary diversity were collected using the WHO indicators for assessing infant and young child feeding practices [2]. The 2011 EDHS questionnaires had parts divided into three; household, woman’s and man’s questionnaires. Generally, the questionnaires capture information on socio-demographic variables, reproductive and other health issues, health service characteristics and use. The woman’s questionnaire had parts for children, which capture information on child immunization, health and nutrition. Similarly, the WHO infant and young child feeding indicators had details on the purpose of each indicator, measuring methods and definitions of each indicator. It had detailed description of dietary diversity, including minimum dietary diversity. The dietary diversity data were collected using a 24-h recall method; that is, mothers were asked to recall all foods given to their child in the past twenty-four hours before the survey.

Pretest was done before the actual data collection outside of the study area. Five data collectors who were health professionals and two supervisors who have had experience in supervision were recruited and trained for two days to facilitate the data collection process. Training was given for data collectors and supervisors on the rationale, objective of the study, confidentiality, the process and technique of data collection. On daily basis, close supervision was made, and feedback and correction was given for the data collectors and supervisors. Furthermore, every day, completeness and consistency of the collected data were checked.

In this study, a minimum dietary diversity was defined as the proportion of children 6–23 months of age who received foods made from four or more food groups out of the seven food groups during the previous day [2]. Moreover, knowledge about dietary diversity and child feeding was measured based on ten knowledge questions. Each correct response (yes) earned one point, whereas any wrong response (no) got zero. The calculated knowledge score ranged from 2 to 10 points. A median score of seven points (±1.55 standard deviation) was used to decide the cutoffs. Based on this, knowledgeable mothers were those who had a score of seven and above from the provided ten knowledge questions about dietary diversity and child feeding (Table 1).

**Table 1:** Knowledge of mothers on dietary diversity and child feeding

Knowledge variables	Frequency (percentage)
Heard about importance of feeding diversified foods to a 6–23 month child	263 (74.7)
Complementary feeding should start at 6 months of child age	325 (92.3)
A 6–23 month child should eat four or more food groups	176 (50.0)
Giving meat is advisable for 6–23 month child	216 (61.4)
One cause of childhood malnutrition is not having diversified foods	316 (89.8)
Didn’t feel hungry doesn’t mean that the nutritional need of a child is fulfilled	263 (74.7)
One cause of childhood malnutrition is not starting complementary feeding at 6 months of child age	308 (87.5)
Feeding only animal products is not enough/adequate for 6–23 month child	278 (79.0)
A 6–23 month child should feed organ meat, like liver, kidney	93 (26.4)
A 6–23 month child should feed egg	331 (94.0)
Overall knowledge score	
Good knowledge	261 (74.1)
Poor knowledge	91 (25.9)

### Data analysis

The collected data were entered into EPI Info 7 and exported to SPSS 24 for further analysis. Both descriptive and analytic statistical methods were used to present the findings of the study. Frequencies and cross tabulation were used to summarize descriptive statistics of the data. A binary logistic regression analysis was run to identify associations between the dependent (minimum dietary diversity score – ‘yes’ versus ‘no’ option) and independent variables (socio-demographic characteristics of children, obstetrics and health service characteristics of mothers, knowledge of mothers on dietary diversity and child feeding, and dietary diversity feeding practices). The dependent variable – ‘minimum dietary diversity score’ was coded as ‘1’ for those who had consumed four or more foods and ‘0’ for less than four food groups during the previous day. Finally, variables with *P*-value <0.05 were included in the multivariable logistic regression model to identify factors independently associated with feeding MDD. The level of significance was determined at P-value of less than 0.05 with a 95% confidence interval. The fitness of the regression model was tested through Hosmer-lemeshow goodness of fit.

## Result

### Socio demographic characteristics

In this study, among the sampled 365 study participants, 352 of them were participated, giving a response rate of 96.4%. The median age of the children was 15 (±5.84 standard deviation) months where the highest proportion of them, 156 (44.3%) belongs to the age group of 6–11 months. Out of the total mothers interviewed, most of them (40.3%) were between the age group of 25–29 years. Almost half of the children were the first child of their parents. The majority of the mothers (94.9%) were married (currently in a union), 221 (62.8%) Orthodox Christians by religion and 113 (32.1%) of the mothers had attained secondary level of education. With regard to occupational status of children’s parents, most of the mothers (69.9%) and only 65 (18.5%) of the fathers were unemployed. Above one third of the households – 118 (33.5%) had a monthly income of between 1501 and 3000 Ethiopian Birr (Table 2).

**Table 2:** Socio-demographic characteristics of children age 6–23 months in Addis Ababa, Ethiopia, 2016 (n = 352)

Variable	Category	Frequency (percentage)
Sex of child	Male	192 (54.5)
	Female	160 (45.5)
Mother’s age	<= 24 years	98 (27.8)
	25–29 years	142 (40.3)
	≥30 years	112 (31.8)
Child’s age	6–11 months	156 (44.3)
	12–17 months	65 (18.5)
	18–23 months	131 (37.2)
Birth order	First	174 (49.4)
	Second	124 (35.2)
	Third and above	54 (15.3)
Mother’s marital status	Married	334 (94.9)
	Not in union^a^	18 (5.1)
Mother’s religion	Orthodox	221 (62.8)
	Protestant	25 (7.1)
	Muslim	106 (30.1)
Mother’s education	No formal education	92 (26.1)
	Primary education	95 (27.0)
	Secondary education	113 (32.1)
	College and above	52 (14.8)
Father’s education	No formal education	35 (9.9)
	Primary education	107 (30.4)
	Secondary education	120 (34.1)
	College and above	90 (25.6)
Mother’s occupation	Unemployed	246 (69.9)
	Government employed	31 (8.8)
	Merchant	40 (11.4)
	Private employed	35 (9.9)
Father’s occupation	Unemployed	65 (18.5)
	Government employed	64 (18.2)
	Merchant	113 (32.1)
	Private employed	110 (31.3)
Household monthly income (Ethiopian Birr)	<1500	102 (29.0)
	1501–3000	118 (33.5)
	3001–4500	52 (14.8)
	>4500	80 (22.7)
Family size	2–3	146 (41.5)
	4–5	171 (48.6)
	> = 6	35 (9.9)

^a^Single/divorced/widowed/separated

### Obstetric characteristics of mothers

Three hundred forty one (96.9%) of the mothers had more than one pregnancy history (gravidity). Concerning parity, 150 (42.6%) of them had one to two live births, whereas 171 (48.6%) and 31 (8.8%) had three to four, and more than four live births, respectively. Three hundred twenty one (92.2%) of the mothers had four or more antenatal care visits for their last pregnancy. Most of them (97.4%) had given their last births at health facilities. Almost all of the mothers (99.4%) had postnatal care (PNC) visits for their last child.

### Knowledge of mothers on dietary diversity and child feeding

Concerning the knowledge of mothers about child feeding, out of the ten knowledge questions on dietary diversity and child feeding almost three fourth of them (74.1%) were knowledgeable answering seven and above questions correctly. Amongst 352 study participants interviewed, 263 (74.7%) of the mothers had heard of feeding diversified food to their children. The main sources of information were health institutions – 180 (68.4%) followed by mass media – 77 (29.3%) and family members 49 (18.6%). Here, most of the mothers (92.3%) knew that complementary foods should be introduced at six months of child age.

With regard to dietary diversity, half of the mothers stated that a child should consume at least four types of food groups. Approximately three fourth of the mothers (74.4%) stated that even if a child didn’t feel hungry, it doesn’t mean that his/her nutritional requirement is fulfilled. Two hundred seventy-eight (79.0%) of the mothers/caregivers recognized that giving only animal source food is not adequate for child growth and development (Table 1).

### Minimum dietary diversity feeding practices

All the children included in this study had started taking solid, semi-solid or soft foods, and all of them had received complementary foods the previous day before the survey. The frequency of child feeding on the 24-h recall was four or more times for 194 (55.1%) of the children’s followed by three times – 124 (35.2%) and one to two times – 34 (9.7%). Mothers reported to have introduced other foods apart from breast milk for the first time before their child reached six months were only 37 (10.5%). On the other hand, majority of the mothers (81.8%) started at six months as per recommended, followed by 27 (7.7%) respondents who had initiated complementary foods to their children after passing six months of age.

**Fig. 1:**
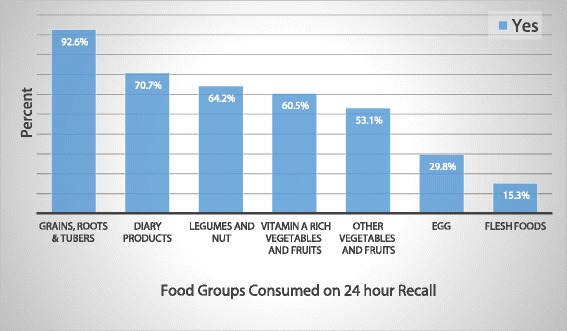
Types of food groups consumed by children aged 6–23 months in Addis Ababa, Ethiopia, 2016 (*n* = 352)

The dietary diversity determined based on a 24-h recall method showed that 59.9% (95% CI: 54.7–65.3) of the children had received minimum dietary diversity. Grain, roots and tubers were the most commonly consumed food items 24 h preceding the survey; i.e., it is consumed by 326 (92.6%) of the children followed by dairy products – 249 (70.7%). However, the intake of flesh food or organ meat was low; of the total children only 54 (15.3%) ate flesh food (Fig. 1).

### Factors associated with feeding minimum dietary diversity

According to the multivariable analysis mother’s education, household income and knowledge on dietary diversity and child feeding were significantly associated with minimum dietary diversity feeding practices. However, mother’s age and occupation, and father’s education and occupation didn’t show any significant association in the multivariable analysis. Moreover, obstetrics and health service characteristics of mothers didn’t show any statistical association at both levels; the binary and multivariable logistic regression model.

The odds of feeding minimum dietary diversity to child’s age 6–23 months was significantly associated and higher among mothers who had attained secondary, and college and above level of education, with an adjusted odds ratio of 4.62 (95% CI: 2.31–9.25) and 4.49 (95% CI: 1.50–13.42) respectively as compared to those who had no formal education. Mother’s good knowledge on dietary diversity and child feeding was significantly associated with the feeding of the minimum dietary diversity, with an adjusted odds ratio of 1.98 (95% CI: 1.11–3.53) to their child. Furthermore, children of mother’s who had a household monthly income of greater than 3000 Ethiopian Birr were more likely to feed the minimum dietary diversity as compared to those from a family of a monthly income <=1500 Ethiopian Birr (Table 3).

**Table 3:** Factors associated with feeding minimum dietary diversity among children age 6–23 months in Addis Ababa, Ethiopia, 2016 (n = 352)

Variable		Minimum Dietary Diversity		Crude Odds Ratio (95% CI)	Adjusted Odds Ratio (95% CI)
		Yes	No		
		(n, %)	(n, %)		
Mother’s age (in years)	<=24	51 (52)	47 48)	1.00	1.00
	25–29	94 (66.2)	48 (33.8)	1.81 (1.07–3.06)*	1.40 (0.73–2.67)
	> = 30	66 (58.9)	46 (41.1)	1.32 (0.77–2.28)	1.26 (0.65–2.45)
Mother’s education	No formal education	26 (28.3)	66 (71.7)	1.00	1.000
	Primary education	51 (53.7)	44 (46.3)	2.92 (1.60–5.40)***	1.83 (0.95–3.53)
	Secondary education	88 (77.9)	25 (22.1)	8.94 (4.74–16.86)***	4.62 (2.31–9.25)***
	College diploma & above	46 (88.5)	6 (11.5)	19.46 (7.42–51.04)***	4.49 (1.50–13.42)**
Mother’s occupation	Unemployed	129 (52.4)	117 (47.6)	1.00	1.00
	Government employed	26 (86.9)	5 (16.1)	4.72 (1.75–12.68)**	0.81 (0.29–3.15)
	Merchant	26 (85.0)	14 (35.0)	1.68 (0.84–3.38)	1.30 (0.56–2.99)
	Private employed	30 (85.7)	5 (14.3)	5.44 (2.04–14.49)**	2.90 (0.97–8.70)
Father’s education	No formal education	11 (31.4)	24 (68.6)	1.00	1.00
	Primary education	44 (41.1)	63 (58.9)	1.52 (0.68–3.43)	0.55 (0.21–1.43)
	Secondary education	80 (66.7)	40 (33.3)	4.36 (1.95–9.79)***	0.75 (0.26–2.14)
	College diploma & above	76 (84.4)	14 (15.6)	11.84 (4.75–29.52)***	1.01 (0.30–3.41)
Father’s occupation	Unemployed	35 (53.8)	30 (46.2)	1.00	1.00
	Government employed	48 (75.0)	16 (25.0)	2.57 (1.22–5.43)*	1.27 (0.48–3.34)
	Merchant	61 (54.0)	52 (46.0)	1.00 (0.55–1.85)	0.91 (0.43–1.91)
	Private employed	67 (60.9)	43 (39.1)	1.34 (0.72–2.48)	0.88 (0.41–1.87)
Household income (Ethiopian Birr)	<=1500	33 (32.4)	69 (67.6)	1.00	1.00
	1501–3000	65 (55.1)	53 (44.9)	2.56 (1.48–4.45)**	1.60 (0.87–2.93)
	3001–4500	40 (76.9)	12 (23.1)	6.97 (3.24–15.01)***	4.13 (1.80–9.50)**
	>4500	73 (91.3)	7 (8.8)	21.81 (9.05–52.54)***	9.21 (3.48–24.38)***
Dietary diversity and child feeding knowledge	Poor	35 (38.5)	56 (61.5)	1.00	1.00
	Good	176 (67.4)	85 (32.6)	3.31 (2.02–5.44)***	1.98 (1.11–3.53)*

*p-value <0.05, ** *p*-value <0.01, ***p-value <0.001

## Discussion

The improvement in infant and young child feeding (IYCF) practices plays a critical role in the improved nutrition, health and development of a child [20] and it is due to this reason that the world health organization recommends the consumption of at least four food groups [2]. In this case if a child fed on, at least from four food groups on the previous day, it is assumed that; in most populations, the child could have a higher probability to consume at least one animal-source food and at least one fruit or vegetable in addition to the usual or staple food items (grain, root or tuber).

In this study, 59.9% (95% CI: 54.7–65.3) of the children aged 6–23 months had fed on four or more food groups meeting the minimum requirement of diversified diet. This finding is similar to a study done in Sri-Lanka [21], however, it is much higher than the reported national 2011 EDHS [12] and some other national studies [13–15]. Furthermore, it is higher than other studies done in Ghana [16], east Delhi, India [17] and Bangladesh [22]. Since this study was done at the urban center – the capital city of Ethiopia, mothers’ might have easy access to information or media and health services about dietary diversity and child feeding practices [12–14]. It might also be due to the difference in the study design and setting. However, this study and the above-mentioned studies were cross-sectional, most of them were community-based studies done in rural areas. In community-based studies, the overall estimate of optimum dietary diversity could be lower than health facility studies, which we noticed in these studies. Moreover, differences in countries setting, the self-reported measurement and recall method could also have an effect on the estimated minimum dietary diversity score.

It was found that maternal educational attainments of secondary and above were significantly associated with minimum dietary diversity compared to those mothers who had no formal education. This finding is consistent with a study done in North West Ethiopia [14], five South Asian countries [23] and Sri-Lanka [21] where mother’s higher educational attainment and overall literacy rate was a significant determinant factor for appropriate diversified infant feeding practices. This could be due to the fact that educated mothers might be more likely to have more information, understood educational messages delivered through different media outlets, engaged in paid works and might learn on child feeding in the curricula at school.

This study also showed that feeding of a child from a diversified food sources is significantly associated with a higher household monthly income. This is supported by Nepalese study – a secondary data analysis of the Nepalese Demographic and Health Survey [24]. The 2011 Ethiopian DHS also reported that children from a family of highest wealth quintile were more likely to fed on four or more food groups [18]. This could be attributed to the fact that children’s from a family of higher monthly income might feed diversified foods as their families could be more likely to afford to have diversified foods as compared to children’s from a low household income.

Moreover, this study has confirmed that appropriate dietary diversity feeding practice is influenced by mothers’ knowledge about dietary diversity and child feeding practices. Mothers, who had a good knowledge about dietary diversity and child feeding practices were more likely to feed their children diversified foods than their counterparts. This finding is supported by a study done in southern Ethiopia that a unit increase in maternal knowledge on IYCF was associated with a 0.41 increase in dietary diversity score [13]. Moreover, children from mothers who had received IYCF message during PNC, participated in food cooking demonstration [13] and exposure to IYCF information on the mass media were more likely to feed on diversified foods [13, 14]. The study done in Nairobi, Kenya [25] supports this finding; that is, mothers knowledge on healthy eating habits is related to a diversified child feeding practices. A better understanding about the diversified food items and its benefits to the health and development of a child is important for appropriate child feeding practices. This could be achieved through the Health Extension Workers – their main role is to teach the community about the sixteen health extension packages (nutrition is amongst these package) [26] through house to house visit and at the health facility besides providing some curative services.

### Limitations of the study

This study shares the common limitation of cross-sectional study – difficult to make causal association. Being a health facility based study; particularly governmental health facilities - ignored the entire community and those who had service in private health facilities, might under or overestimate the finding. In addition to this, as the study considered only 24-h recall method, it might not accurately reflect participants past feeding dietary habit. Moreover, there might be a recall bias, and being a self-reported study might not give the exact figure of the minimum dietary diversity practice. Even though those children who were sick were excluded, there could be some children who were not sick on the previous one week, but lost their appetite, which could underestimate our finding.

## Conclusion

In this study, the consumption of minimum dietary diversity among children aged between 6 and 23 months was found to be high. Even though this study showed a better result, it still needs more effort to achieve the recommended minimum dietary diversity intake for all children aged between 6 and 23 months. Educational status of the mother, household monthly income and knowledge of the mother on diversified foods were the factors which were found to be positively associated with providing the minimum dietary diversity. Children from a low socioeconomic status and mothers with no formal educational attainment needs special attention to improve the practice of appropriate feeding of young children.

## Abbreviations

AOR: Adjusted Odds Ratio
CI: Confidence Interval
COR: Crude Odds Ratio
DD: Dietary Diversity
EDHS: Ethiopian Demographic and Health Survey
EPI: Expanded Program on Immunization
IYCF: Infant and Young Child Feeding
MCH: Maternal and Child Health
MDD: Minimum Dietary Diversity
PNC: Postnatal Care
WHO: World Health Organization
